# Relationship between Pharmacokinetic Profile and Clinical Efficacy Data of Three Different Forms of Locally Applied Flurbiprofen in the Mouth/Throat

**DOI:** 10.3390/pharmaceutics15071863

**Published:** 2023-07-01

**Authors:** Vit Perlik, Anuradha Kulasekaran, Graça Coutinho, Martin Votava, Jean-Michel Cardot

**Affiliations:** 1 Institute of Pharmacology, First Faculty of Medicine, Charles University, Albertov 4, 12800 Prague, Czech Republic; vitperlik@gmail.com; 2 Reckitt Healthcare Limited, 103-105 Bath Road, Slough, Berkshire SL1 3UH, UK; graca.coutinho@reckitt.com; 3 Cadore INV Scientific Consultant, Akademicka 1, 10800 Prague, Czech Republic; martin.votava@cadoreinv.com; 4 SAS BORVO Consultant, 18 Av de Charade, 63122 Ceyrat, France; j-m.cardot@orange.fr

**Keywords:** locally applied locally acting, LALA, GIT, lozenge, throat, flurbiprofen, pharmacokinetics, pharmacodynamic

## Abstract

This study aimed to link pharmacokinetic (PK) data from different flurbiprofen preparations for the treatment of sore throat with published data to elucidate whether early efficacy is due to the local action of flurbiprofen or a systemic effect after absorption of the swallowed drug. Three comparative bioavailability studies conducted in healthy subjects provided data from flurbiprofen 8.75 mg formulations, including spray solution, spray gel, lozenges, and granules. A parallel interstudy comparison was made of PK parameters, including partial AUCs (pAUCs), using an ANOVA model with the calculation of 90% confidence intervals (CI) for the differences between least squares (LS) means for each of the test groups versus the respective reference groups. All three studies showed bioequivalence for the respective product comparisons. The interstudy comparison showed a slower rate of absorption for granules compared to spray solution (reference) based on T_max_, C_max_, and pAUCs for 1 h and 2 h. When AUC_0.25h_ and AUC_0.5h_ were considered, slower rates of absorption were also seen for lozenges and spray gel. The differences correlated with the reported time of onset of action, which is faster for the spray solution (20 min) compared to lozenges (26 min) and granules (30 min). These pAUCs provide useful data that allow for the discrimination between formulations. Moreover, the pAUC values represent <5% of the total AUC, suggesting that the early onset of pain relief is a response to immediate local absorption at the site of action rather than a systemic effect.

## 1. Introduction

The main objective of developing locally applied products, including non-steroidal anti-inflammatory drugs (NSAIDs), is to ensure that they are delivered locally and exert their effect only at the locally affected site, with any systemic effects being considered undesirable [1,2]. The site-specific absorption of locally applied NSAIDs has been achieved through targeted delivery using various pharmaceutical forms with evidence of local tissue concentration [3,4]. This maximises the local effect of NSAIDs at the site of inflammation while reducing the dose administered to the patient in order to limit systemic exposure and thus potential adverse effects [5,6]. The concept of local delivery of a low dose of the drug for localised effect has been applied successfully to several NSAIDs [7], with efficacy having been demonstrated despite much lower systemic exposure compared with oral administration.

For example, in a study in healthy subjects that compared topical diclofenac sodium 2% solution twice daily and diclofenac sodium 1.5% solution four times daily to oral diclofenac sodium 75 mg tablets twice daily, the 2% and 1.5% topical solutions were bioequivalent with respect to AUC at steady state, with approximately 93% lower systemic exposure compared to oral administration [8]. Topical application was associated with slower absorption and delayed elimination, with an apparent terminal half-life 4 to 6 times longer than that for oral diclofenac. This is presumed to be due to a reservoir effect in the skin and subcutaneous fat, from which there is a sustained release of drug into the underlying target tissues [9]. The much lower exposure seen with the topical solutions did not affect efficacy when compared with oral diclofenac but was associated with significantly fewer adverse events such as gastrointestinal (GI) complaints and increased liver enzymes [3,10,11].

The efficacy of locally applied, locally acting NSAID products is a consequence of higher concentrations achieved in the target tissues when compared to those seen in the systemic circulation. Topical diclofenac or flurbiprofen, for example, are associated with better penetration [5,12] and permeation of the drug into the target tissues, with a lower plasma-to-tissue ratio than is seen for oral diclofenac [9]. Following application of diclofenac sodium 4% spray gel to the knee two- or three-times daily for 3 days prior to planned total arthroplasty, the median diclofenac concentration was approximately 10–20-fold higher in synovial tissue (36.2 and 42.8 ng/g) than in synovial fluid (2.6 and 2.8 ng/mL) or plasma (3.9 and 4.1 ng/mL) [3]. Moreover, after topical delivery, diclofenac is highly bound to the target tissues, so levels are sustained for a longer duration (at least several hours) compared to oral administration, thus maintaining concentrations that are sufficient to exert a therapeutic effect [9].

Similar data have been reported for flurbiprofen. For example, in patients administered flurbiprofen 40 mg perorally or topically 16 h and 2 h prior to knee surgery, significantly higher concentrations of the active drug were found in the fat, tendon, muscle, and periosteal tissues in the topical application group compared to those who received tablets [12].

Although the efficacy and safety of locally applied NSAIDs are well established in a variety of indications, the role of local versus systemic concentrations in the pharmacological effects remains incompletely understood. For flurbiprofen 8.75 mg lozenges and sprays used in the treatment of oral inflammatory conditions, in vitro data show that the active drug reached all layers of the human cadaveric pharynx mucosal tissue, including the underlying lamina propria, which contains blood vessels and nerve fibres that contribute to pain during pharyngitis [4,13]. This suggests that flurbiprofen has a local mechanism of action for sore throats that is linked with the concentration in the oral tissues rather than systemic blood concentrations. An adhesive gel spray formulation that has shown, in vitro, higher penetration into the oropharyngeal tissues could, potentially, have better targeted clinical efficacy and reduced the scope for adverse events by reducing the amount of swallowed flurbiprofen (data on file). This is consistent with published data for different topical pharmaceutical forms of flurbiprofen (solution, granules, and lozenges), which differ with respect to onset of action (from 2 to 5 min) and time to meaningful pain relief [14,15,16].

In this study, we compared the PK parameter (pAUC) of different locally applied flurbiprofen preparations with the efficacy parameter (time to clinically meaningful pain relief) from published studies of the treatment of sore throat. Such a comparison contributes to the elucidation of whether initial efficacy is due to local absorption in the throat rather than systemic absorption of swallowed flurbiprofen from the GI tract.

## 2. Materials and Methods

### 2.1. Pharmacokinetic Studies

Three randomised, open-label, crossover design PK studies were conducted in healthy male and female subjects following standard inclusion/exclusion criteria for bioavailability and bioequivalence studies [17,18], including an age range of 18–55 years with normal oral mucosa and a Body Mass Index (BMI) of 18.5–30 kg/m^2^. The studies were conducted in accordance with the Declaration of Helsinki and Good Clinical Practice recommendations. Approvals were obtained from an ethics committee in line with local regulations, and written informed consent was obtained from each participant. The studies were registered on the European Union Drug Regulating Authorities Clinical Trials Database (EudraCT).

Study EudraCT 2018-003175-36 included 16 subjects and compared two flurbiprofen viscous spray gel formulations to a reference spray solution (non-viscous). The treatment regimen for all products was a single therapeutic dose of 3 sprays (equivalent to 8.75 mg flurbiprofen) delivered to the back of the throat.

The study EudraCT 2011-003332-31 compared two spray solutions (Treatments B and D; 3 sprays for an 8.75 mg dose) to a flurbiprofen 8.75 mg lozenge formulation (Treatment A) in 33 subjects.

Finally, the randomised study EudraCT 2008-005177-34 consisted of 16 subjects using flurbiprofen granules or a lozenge formulation, both at a strength of 8.75 mg, crossing over to the alternative treatment in the second period.

A total of 12, 18, and 15 blood samples, respectively, were drawn over 720 min from each subject at each study period in the first, second, and third studies; all studies included sampling at least every 5 min for the first 15 min (two studies involving spray also included a 2-min sample), along with further sampling at 30 min. The sampling time points were based on standard requirements of adequate sampling prior to and around the C_max_ [1] and then up to 720 min to capture the full plasma concentration profiles up to more than 3 terminal half-lives. The spray studies included an earlier sampling time point based on a pilot study (Study No. TH0918 [19]). The plasma obtained was analysed for flurbiprofen using a validated high-performance liquid chromatography-tandem mass spectrometry (HPLC-MS/MS) method [20,21]. The following PK parameters were derived to describe the PK properties of the respective flurbiprofen formulations and are similar to those published for the spray solution pilot study (Study No. TH0918 [19]): maximum observed plasma concentration (C_max_), area under plasma concentration curve from administration to last quantifiable concentration at time t (AUC_0−t_), time to maximum observed concentration (T_max_), AUC extrapolated to infinity (AUC_0−inf_), elimination rate constant (K_el_), and elimination half-life (T_½_). An Analysis of Variance (ANOVA) model (separate models for each product) using Excel was fitted to naturally log-transformed (ln) AUC_0−t_, C_max_, and AUC_0−inf_ with fixed terms for treatment, period, sequence, and subject nested within sequence, and 90% CI for the differences between LS means for each of the test groups versus the chosen reference group were calculated for each of the individual PK studies.

### 2.2. Parallel Interstudy Comparison and Partial AUCs

Plasma concentration data from all three PK studies was combined into one database. Partial AUCs (pAUCs) over the first two hours after administration of flurbiprofen products were calculated using the linear trapezoidal rule as an additional metric to reflect the rate of absorption [22,23]. The pAUCs up to 30 min also serve as a tool in the case of locally applied products to separate early drug absorption at the site of action from the systemic absorption of swallowed drugs from the GI tract [1]. The parallel interstudy comparison was based on an ANOVA model that was fitted to ln AUC0-t, Cmax, and pAUCs (for the intervals 0–15 min, 0–30 min, 0–1 h, and 0–2 h) with fixed terms for treatment, and the 90% CI for the differences between LS means for each of the test groups versus the respective reference groups was calculated. Reference scaling was used in order to take potential differences in study design into account in the across-study comparison, as previously described by Cardot et al. [24]. Data from spray formulations and subsequently from the flurbiprofen lozenge formulation were used for this purpose, both representing common points across the PK studies. Finally, linear regressions were used to compare the reference scaled early pAUCs (0–15 and 0–30 min) data from the studies with the respective onset of action times previously reported for different formulations [14,15,16,25]. Specifically, time to clinically meaningful pain relief [25,26] was used in order to provide a comprehensive treatment comparison with direct clinical implications.

## 3. Results

**Figure 1:**
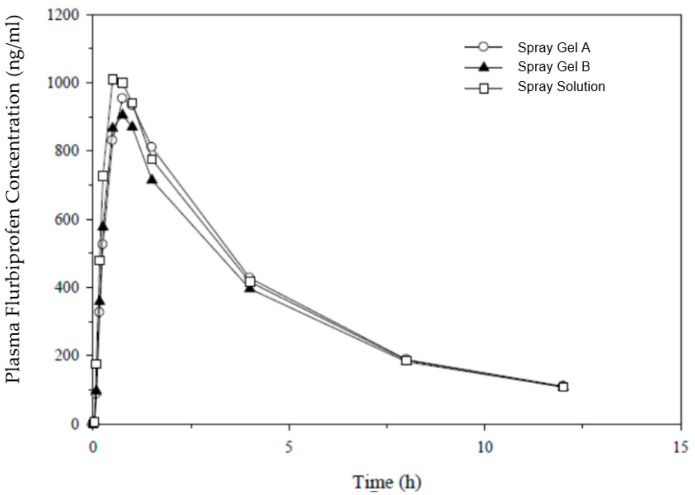
Plasma concentrations after a single therapeutic dose of flurbiprofen (8.75 mg) delivered to the back of the throat in the pharmaceutical form of spray gel (A and B) and a reference spray solution.

The results of the study EudraCT 2018-003175-36 in 16 subjects are presented in Figure 1 and demonstrate a similar overall exposure for all spray formulations (simple solution and gel).

The PK parameters were similar for extent of absorption, with geometric means for AUC_0−t_ ranging from 3930 ng∗h/mL (spray gel B) to 4225 ng∗h/mL (marketed spray solution), denoting a 7% difference between the extremes. As a measure of rate of absorption, the geometric means for C_max_ ranged from 922 to 1040 ng/mL (highest for the marketed spray), with a 12% difference between the extremes. Both spray gel formulations were bioequivalent to the reference spray solution product, with 90% CI for AUC and C_max_ falling within the standard acceptance range of 80–125%, apart from the lower CI for C_max_ for the second spray gel formulation (B), which was marginally below 80%. No statistically significant differences were observed in secondary PK parameters (T_max_, T_½_, and K_el_) when the new spray gel formulations were compared with marketed spray solutions.

**Figure 2:**
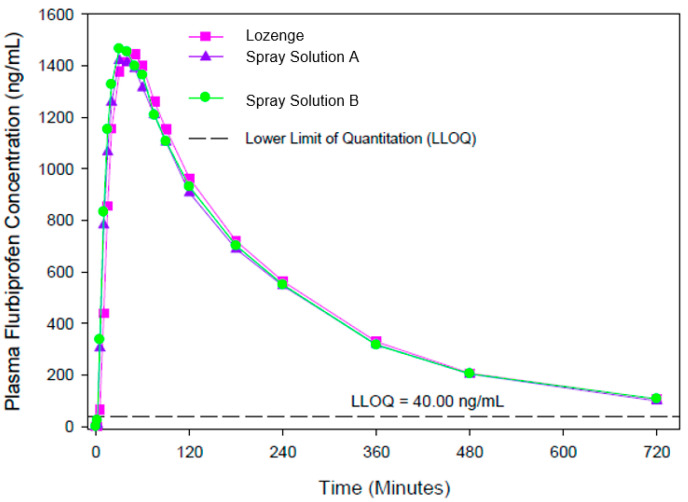
Plasma concentrations after a single therapeutic dose of flurbiprofen (8.75 mg) delivered to the back of the throat in the pharmaceutical form of spray solutions (A and B) and lozenges.

The results of study EudraCT 2011-003332-31 in 33 subjects (presented in Figure 2) also demonstrated similar overall exposure for all formulations (8.75 mg spray solution and lozenges).

The spray solutions (Treatments B and D) and lozenges (Treatment A) differed by less than 3% for extent of absorption (AUC_0−t_ geometric means ranging from 5544 to 5682 ng∗h/mL) and by less than 2% for rate of absorption (C_max_ geometric means ranging from 1553 to 1580 ng/mL). The 90% CIs for the ratios of the geometric means for C_max_ and AUC_0−t_ fell within the standard acceptance range of 80–125%, confirming the bioequivalence of the spray solutions compared to the lozenges. There was a statistically significant difference between T_max_ values (Wilcoxon Matched Pair Test; *p* value = 0.030, D versus A), with plasma concentrations peaking earlier for the spray solutions (median 0.50 h) than for the lozenges (median 0.83 h), a difference of well over 20%.

**Figure 3:**
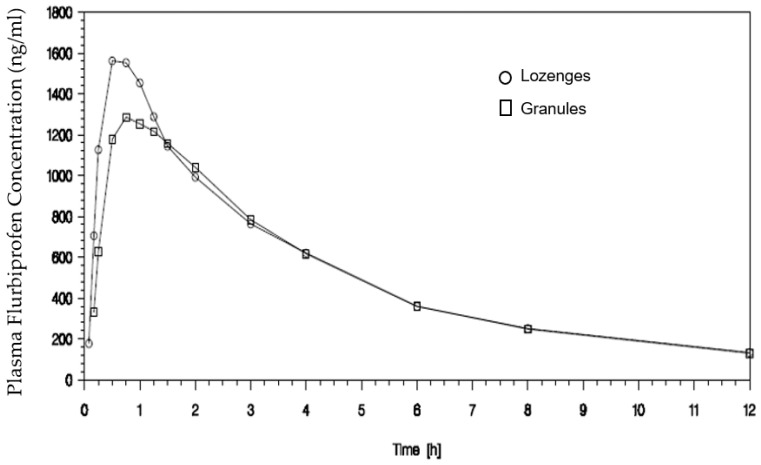
Plasma concentrations after a single therapeutic dose of flurbiprofen (8.75 mg) delivered in the mouth in the pharmaceutical form of granules and lozenges.

Finally, study EudraCT 2008-005177-34 in 16 subjects also demonstrated a similar overall exposure for both formulations (granules and lozenges), as shown in Figure 3.

The extent of absorption (AUC_0−t_) was similar for granules and lozenges, with geometric means (5932 and 6251 ng∗h/mL) differing by approximately 5%. For C_max_ as a measure of rate of absorption, the respective geometric means were 1413 and 1620 ng/mL, just less than 13% lower for granules compared to lozenges. The formulations were bioequivalent, with 90% CIs for the ratios of geometric means for C_max_ and AUC_0−t_ falling within the standard acceptance range of 80–125%. There was a statistically significant difference between formulations for peak plasma concentrations (Wilcoxon Matched Pair Test; *p* value = 0.030); although median T_max_ values for the lozenge formulation (0.75 h) differed from granules (0.88 h) by less than 20%, the respective ranges differed markedly (0.50 to 1.00 h for lozenges and 0.25 to 2.00 h for granules), and the difference between arithmetic mean T_max_ values was ~25 min.

### 3.1. Sensitivity Analysis

The parallel interstudy comparison confirmed the bioequivalence of the spray gel, lozenges, and granules to the marketed spray solution formulation (reference value, 100%) with respect to extent of absorption (AUC_0−t_) but suggested possible differences for rate of absorption as measured by C_max_, with a slower rise in plasma levels for granules when compared to spray solution (Table 1).

### 3.2. Partial AUC Comparison

The suggested differences in the formulations are not clearly illustrated using traditional metrics as applied to the assessment of bioequivalence between formulations. Therefore, additional post hoc analyses were performed in order to describe the early phase of the absorption process and the initial onset of measurable plasma levels. Individual plasma concentration data were used to calculate pAUCs for the intervals 0–15 min, 0–30 min, 0–1 h, and 0–2 h (Table 2 and Table 3).

The pAUCs at these earlier time points more clearly indicate possible differences between the formulations. As shown in Table 2 and Table 3, the analysis revealed statistically significant differences between the granule formulation and spray solution for AUC_0.25h_ and AUC_0.5h_, and also for AUC_1h_ and AUC_2h_. For the comparison between lozenges and the spray solution, a significant difference was seen only for AUC_0.25h_, with a trend for a difference for AUC_0.5h_ (*p* value = 0.13). A similar pattern was also observed for the comparison between one of the spray gel formulations (A) and the spray solution, with a significant difference detected for AUC_0.25h_ and a trend for a difference for AUC_0.5h_ (*p* value = 0.08). The behaviour of the spray gel formulation (B) was not statistically different from the spray solution for any of the pAUCs.

### 3.3. Correlation of Early Partial AUCs with the Onset of Action

In order to elucidate the possible links between therapeutic effect and early exposure, the pAUCs for 0–15 min and 0–30 min were compared and correlated with the previously published onset of action data, specifically the time to clinically meaningful pain relief for the different formulations (Table 4).

Linear correlation coefficient values were close to 1, strongly supporting a link between the extent of absorption of flurbiprofen in the first 15–30 min and the timing of onset of action (time to clinically meaningful pain relief) as reported in previously published therapeutic trials [16,25].

## 4. Discussion

Currently, flurbiprofen is the only low-dose NSAID [14,15] for oromucosal drug delivery that can be used for the treatment of sore throat globally, including the EU, Russia, LATAM, and certain ASEAN regions. The use of locally applied, locally acting NSAIDs is preferred due to a lower frequency of adverse effects when compared to systemic NSAID treatment [27]. Sore throat constitutes a significant burden on quality of life even within a short period of time [28], and so it is important to have products on the market that have a rapid onset of action and a comparable level of efficacy to classical peroral NSAIDs. The rapid onset of action of analgesic formulations generally provides better overall pain relief and a lesser need for additional analgesia [29].

The PK data presented above show that locally acting flurbiprofen 8.75 mg formulations, including sprays (simple solution and gel spray), lozenges, and granules, all exhibit a very similar extent of absorption as shown by similar (bioequivalence criteria, defined as 90% CI within the standard range of 80–125%) AUC_0−t_ values. Moreover, the rate of absorption as expressed by C_max_ values is also similar, with a 90% CI within or close to the standard bioequivalence range of 80–125%, albeit with slightly slower absorption for granules when compared to lozenges (Cmax value lower by about 13%; later T_max_). In contrast, it is known that different topical pharmaceutical forms of flurbiprofen (spray solution (Study No. TH0918 [19]), lozenges, and granules) differ in terms of time to onset of action, a clinically relevant efficacy parameter [25]. The available data indicate that patients experience clinically meaningful pain relief around 20 min after using flurbiprofen spray solution and after about 30 min with flurbiprofen granules, while flurbiprofen lozenges fall somewhere between the two (approximately 26 min) [14,15,16,25]. Even when the differences in design of therapeutic trails are taken into account, it seems clear that the nature of the locally applied flurbiprofen formulation impacts speed of effect via differences in local bioavailability identified based on early pAUC_0.25h_ and pAUC_0.5h_.

Given the differences in time to onset of clinically meaningful pain relief for the different formulations that met bioequivalence criteria (90% CI within 80–125%) (Table 1), conventional PK parameters, i.e., AUC_0−t_ and C_max_, do not directly and strongly reflect comparable efficacy [30] for these locally applied, locally acting NSAIDs. Our alternative approach, using reference scaling of data from all PK studies [24], created a data set that allowed comparisons of pAUC correlations to the onset of clinically meaningful effects across the different pharmaceutical forms of flurbiprofen (Table 4).

Thereafter, the formulations were compared based on pAUC calculations for 1 and 2 h (Table 2). These pAUC comparisons indicated statistically significant differences between granules and spray solution, in line with the later T_max_ and lower C_max_ for the granule formulation described above. In contrast, for lozenges vs. spray solution, the 90% confidence intervals for AUC_1h_ and AUC_2h_ fell within standard bioequivalence limits, again suggesting no clinically relevant difference between formulations. This finding is consistent with clinical observations made in therapeutic non-inferiority studies, which demonstrated similar efficacy for these respective formulations at 1 h [31,32]. The pAUCs also show that a newly developed spray gel formulation has a similar pattern of absorption to lozenges, with a point estimate for AUC_1h_ and AUC_2h_ within the standard bioequivalence limits and a lower limit of the 90% CI slightly below the lower acceptance range when compared to spray solution (Table 2), potentially suggesting a lower contribution of GI absorption.

In order to filter out potential GI absorption with resultant systemic effects, pAUCs after 15 and 30 min were also calculated. These early pAUCs most likely reflect local absorption in the oral cavity and pharynx prior to the time when GI absorption could occur and so better reflect permeation into local tissues than AUC data from later timepoints, thus enabling a better description of the behaviour of different formulations of locally applied drugs in the oral cavity and pharynx. A comparison of AUC_0.25h_ and AUC_0.5h_ values for the different formulations revealed significant differences for granules and for lozenges when compared to the spray solution (Table 3), although the AUC_0.5h_ comparison for lozenges with respect to the spray solution did not quite reach statistical significance. On the other hand, when both were compared to the spray solution, the new spray gel formulation outperformed the lozenges with respect to early absorption (AUC_0.25h_) and had similar AUC_0.5h_ values (Table 3). These findings are consistent with the objective for the development of the spray gel formulation, which is designed to have better contact with the mucosa, leading to more targeted delivery and increased residence time within the pharynx, the intended site of action. Such findings have clinical significance since the early onset of action of NSAIDs strongly correlates with their effectiveness [29,33].

The overall PK rank order of the formulations fits well with the formulation properties, with the lowest values observed for granules, followed by lozenges and the spray gel formulation when compared to spray solution. Flurbiprofen granules first need to be dissolved in saliva before absorption can occur; however, once dissolved, the drug will be swallowed rapidly with saliva, leading to a short residence time in the mouth, as shown by the lowest values for partial AUC_0.25h_, AUC_0.5h_, and AUC_1h_ (Table 2 and Table 3). Lozenges contain the solubilised drug in the lozenge mass and provide already dissolved flurbiprofen, which must be released from the formulation by saliva; however, as for granules, the drug will also be partly swallowed, as can be seen from the pAUC analyses (Table 2 and Table 3). The spray solution formulation eliminates the need for dissolution and release from the formulation and provides targeted pharynx delivery, and therefore demonstrated the fastest regional absorption based on all pAUCs. The spray gel formulation was developed to maintain the properties of the spray solution while also extending the regional residence time. As demonstrated, the spray gel, in particular formulation B, provides rapid local delivery as shown by AUC_0.25h_ and AUC_0.5h_ values, as well as sustained regional delivery over the first two hours based on AUC_1h_ and AUC_2h_ values (Table 2 and Table 3). Based on the pAUC data, we think that the differences in formulations are likely to be due to the formulation technology. The gel spray, in contrast to lozenges, is directly applied to the throat but is a more viscous form than the spray solution. The gel spray in contact with the mucosa delivers its active ingredient slower than the spray solution but faster than the lozenges at initial time points, leading to a better performance up to 0.25 h compared to the lozenges, after which it is similar (0.5 h up to 2 h). The viscosity of the gel intentionally limits its capacity to deliver the product rapidly compared to spray solutions, leading to a longer and more constant release closer to that seen for lozenges.

Finally, to better understand the potential link between these earlier pAUCs and the onset of action, a correlation was performed between the absolute values of AUC_0.25h_ and AUC_0.5h_ for all three pharmaceutical forms and clinical efficacy data from published literature [25]. This analysis showed a very clear and strong correlation between early pAUC values and the onset of action (Table 4). Thus, these early partial AUCs (AUC_0.25h_ and AUC_0.5h_), in contrast to later values (AUC_1h_ and AUC_2h_), distinguish between formulations (sprays, lozenges, and granules) that have a similar overall extent of absorption and similar peak plasma concentrations but have been shown to have different times to onset of action in therapeutic trials. The fact that these AUC_0.25h_ and AUC_0.5h_ values represent no more than 1 to 5% of the total AUC suggests that the early onset of pain relief is a response to immediate local absorption at the site of action rather than a systemic effect. This, in turn, would support the use of early pAUCs for clinical effect correlation of locally applied, locally acting products and for head-to-head comparisons, establishing the bioequivalence of different formulations.

Partial AUCs are already currently used by some agencies to better characterise the PK profiles of certain products. For instance, the FDA recommends the use of pAUC as an exposure measure in a number of product-specific bioequivalence guidelines, mainly for certain modified-release (MR) products in which the different phases of release correspond to a clinical effect [18]. European guidelines on bioequivalence of MR products mention the need to include additional parameters, including initial and terminal pAUCs, particularly when a low extent of accumulation is expected [17]. Recently, the importance of the use of pAUC for MR products has been shown by Soares et al. [34], who evaluated 117 studies of prolonged-release products already approved by the Brazilian authority (ANVISA) and found that 24 (20%) failed to demonstrate bioequivalence for the relevant pAUC parameter. Partial AUCs are, under certain circumstances, also a recognised and established metric for treatment comparisons for orally inhaled products [35]. Recently, the draft ICH M13A guideline [22] pointed out that in some situations, C_max_ and AUC_(0−t)_ may be insufficient to adequately assess bioequivalence between two products, in particular when early onset of action is clinically relevant. In these cases, partial AUC may be applied, most typically from the time of drug administration until a predetermined time point that is related to a clinically relevant pharmacodynamic (PD) measure.

The data presented above show that for certain products, such as locally applied, locally acting NSAIDs for use in the oral cavity and throat, pAUCs provide useful data that allow discrimination between formulations. Moreover, these data may be more clinically relevant than those gained through, e.g., charcoal blocks.

It is acknowledged that the exact PK/PD profile of locally applied flurbiprofen has not been reliably established as the presented work is based on parallel study comparisons and retrospective data analyses. The link between PK, local concentrations, and onset of action should be further elucidated in a single study in order to confirm the potential use of early pAUCs as a surrogate for comparison of early pain relief in products of this type.

## 5. Conclusions

While the ultimate proof of similarity across different formulations of locally applied and locally acting drugs in the mouth and throat usually requires therapeutic studies, the comparison of pAUCs provides a promising alternative. The possibility of using PK data, in particular pAUCs, with associated PD correlations to assess the therapeutic equivalence of two formulations containing similar doses of active for local application and local activity in the mouth and throat should be reflected in the equivalence guidelines.

## 6. Patents

The information on gel spray has been submitted for a patent, and further details on this patented formulation are not available for this manuscript.

## Figures and Tables

**Table 1:** Parallel interstudy comparison of the AUC_0−t_ and C_max_ of three pharmaceutical forms of flurbiprofen after a single therapeutic dose with the reference values of the spray solution.

Variable	Test Product (8.75 mg)	Ratio vs. Reference Value %	90% CI	*p* Value
Ln(C_max_)	Lozenges	98.91	86.78–112.75	0.89
	Granules	86.31	75.72–98.39	0.06
	Spray gel A	91.69	80.44–104.51	0.87
	Spray gel B	89.41	78.44–101.92	0.93
Ln(AUC_0−t_)	Lozenges	101.61	89.71–115.10	0.83
	Granules	96.48	85.18–109.28	0.63
	Spray gel A	97.33	85.93–110.25	0.81
	Spray gel B	93.06	82.16–105.40	0.89

**Table 2:** Parallel interstudy comparison of the AUC_1h_ and AUC_2h_ of three pharmaceutical forms of flurbiprofen after a single therapeutic dose with the reference values of the spray solution.

Variable	Test Product 8.75 mg	Ratio vs. Reference Value %	90% CI	*p* Value
Ln(AUC_2h_)	Lozenges	98.95	87.66–111.69	0.89
	Granules	83.25	73.76–93.97	0.01
	Spray gel A	92.12	81.61–103.98	0.87
	Spray gel B	88.55	78.46–99.95	0.87
Ln(AUC_1h_)	Lozenges	93.83	80.58–109.26	0.49
	Granules	65.49	56.24–76.26	>0.001
	Spray gel A	84.12	72.24–97.96	0.42
	Spray gel B	85,54	73.45–99.61	0.85

**Table 3:** Parallel interstudy comparison of the AUC_0.25h_ and AUC_0.5h_ of three pharmaceutical forms of flurbiprofen after a single therapeutic dose with the reference values of the spray solution.

Variable	Test Product 8.75 mg	Ratio vs. Reference Value %	90% CI	*p* Value
Ln(AUC_0.25h_)	Lozenges	59.86	43.99–81.47	0.01
	Granules	25.65	18.85–34.91	>0.001
	Spray gel A	65.18	47.89–88.70	0.002
	Spray gel B	73.60	54.08–100.17	0.86
Ln(AUC_0.5h_)	Lozenges	82.40	66.81–101,63	0.13
	Granules	46.52	37.72–57.38	>0.001
	Spray gel A	73.27	59.40–90.36	0.08
	Spray gel B	79.88	64.77–98.52	0.85

**Table 4:** Parallel interstudy comparison of the AUC_0.25h_ and AUC_0.5h_ of the respective pharmaceutical forms of flurbiprofen and their correlation with efficacy parameter onset of action, specifically time to clinically meaningful pain relief [25].

Variable	Test Product 8.75 mg	pAUC Mean (ng∗h/mL)	Onset of Action (min)	Correlation Coefficient
Ln(AUC_0.25h_)	Flurbiprofen spray solution	84.6799375	20	0.99993962
	Flurbiprofen lozenges	48.1690717	26	
	Flurbiprofen granules	24.5129494	30	
Ln(AUC_0.5h_)	Flurbiprofen spray solution	301.695563	20	0.94568955
	Flurbiprofen lozenges	242.743302	26	
	Flurbiprofen granules	152.584266	30	

## Data Availability

Data are available on request for scientific reasons.
